# Impact of multi-gene mutational profiling on clinical trial outcomes in metastatic breast cancer

**DOI:** 10.1007/s10549-017-4580-2

**Published:** 2017-11-24

**Authors:** Rossanna C. Pezo, Tom W. Chen, Hal K. Berman, Anna M. Mulligan, Albiruni A. Razak, Lillian L. Siu, David W. Cescon, Eitan Amir, Christine Elser, David G. Warr, Srikala S. Sridhar, Celeste Yu, Lisa Wang, Tracy L. Stockley, Suzanne Kamel-Reid, Philippe L. Bedard

**Affiliations:** 1 Division of Medical Oncology and Hematology, Princess Margaret Cancer Centre, 7-723 700 University Avenue, Toronto, Canada; 2 Department of Medicine, University of Toronto, Toronto, Canada; 3 Department of Oncology, National Taiwan University Hospital, Taipei, Taiwan; 4 Laboratory Medicine Program, University Health Network, Toronto, Canada; 5 Department of Laboratory Medicine and Pathobiology, University of Toronto, Toronto, Canada; 6 Cancer Genomics Program, Princess Margaret Cancer Centre, Toronto, Canada; 7 Department of Biostatistics, Princess Margaret Cancer Centre, Toronto, Canada; 8 Department of Medical Biophysics, University of Toronto, Toronto, Canada; 9 Present Address: Division of Medical Oncology and Hematology, Sunnybrook Odette Cancer Centre, Toronto, Canada

**Keywords:** Molecular profiling, Metastatic breast cancer, Targeted therapies, *PIK3CA* mutation

## Abstract

**Purpose:**

Next-generation sequencing (NGS) has identified recurrent genomic alterations in metastatic breast cancer (MBC); however, the clinical utility of incorporating routine sequencing to guide treatment decisions in this setting is unclear. We examine the frequency of genomic alterations in MBC patients from academic and community hospitals and correlate with clinical outcomes.

**Methods:**

MBC patients with good performance status were prospectively recruited at the Princess Margaret Cancer Centre (PM) in Canada. Molecular profiling on DNA extracted from FFPE archival tissues was performed on the Sequenom MassArray platform or the TruSeq Amplicon Cancer Panel (TSACP) on the MiSeq platform. Clinical trial outcomes by RECIST 1.1 and time on treatment were reviewed retrospectively.

**Results:**

From January 2012 to November 2015, 483 MBC patients were enrolled and 440 were genotyped. At least one somatic mutation was identified in 46% of patients, most commonly in *PIK3CA* (28%) or *TP53* (13%). Of 203 patients with ≥ 1 mutation(s), 15% were treated on genotype-matched and 9% on non-matched trials. There was no significant difference for median time on treatment for patients treated on matched vs. non-matched therapies (3.6 vs. 3.8 months; *p* = 0.89).

**Conclusions:**

This study provides real-world outcomes on hotspot genotyping and small targeted panel sequencing of MBC patients from academic and community settings. Few patients were matched to clinical trials with targeted therapies. More comprehensive profiling and improved access to clinical trials may increase therapeutic options for patients with actionable mutations. Further studies are needed to evaluate if this approach leads to improved clinical outcomes.

**Electronic supplementary material:**

The online version of this article (10.1007/s10549-017-4580-2) contains supplementary material, which is available to authorized users.

## Introduction

Mutational profiling of advanced solid tumors is an important component of early phase clinical trials testing drugs in molecularly defined patient populations. This approach, which currently involves the application of next-generation sequencing (NGS) technologies, is used to match a specific therapy to the particular somatic molecular alteration within a patient’s tumor [1]. Although there is a variety of approved systemic therapies for metastatic breast cancer (MBC) that prolong progression-free or overall survival, including endocrine therapy, chemotherapy, and HER2-targeted therapy, MBC is an incurable disease and a leading cause of cancer death worldwide. The identification of subsets of breast cancers with overexpression of the *HER*-*2*/*neu* oncogene by IHC or amplification by fluorescence in situ hybridization (FISH) has led to the addition of targeted therapy for this subtype and improved survival [2, 3]. Additional molecular alterations such as somatic *PIK3CA* mutations, part of the PI3K/AKT/mTOR pathway, are commonly identified in breast cancers but are not yet used in the selection of approved therapies [4–7].

Integrated Molecular Profiling in Advanced Cancers Trial (IMPACT) and our community hospital program Community Oncology Molecular Profiling in Advanced Cancers Trial (COMPACT) were studies at Princess Margaret Cancer Centre (PM) to provide molecular profiling data for advanced solid tumor patients treated at PM and local community hospitals [8]. In this current study, we focus on molecular profiling in advanced breast cancers beyond standard estrogen receptor (ER), progesterone receptor (PR), and HER2 testing. We report on clinical characteristics, somatic mutation frequency, and therapeutic outcomes on genotype-matched and unmatched trials for MBC patients undergoing molecular sequencing of archival tumor tissues.

## Patients and methods

### Study population

Patients with histologically confirmed MBC were eligible for IMPACT/COMPACT if they were ≥ 18 years, had Eastern Cooperative Oncology Group (ECOG) performance status ≤ 1, and had available formalin-fixed paraffin-embedded (FFPE) archival tumor tissue (from either a primary or a metastatic site). This study was approved by the University Health Network Research Ethics Board and was registered on ClinicalTrials.gov [NCT01505400]. Enrollment for IMPACT began in March 2012 and for COMPACT in November 2012 and accrual to IMPACT/COMPACT ended in November 2015. For patients enrolled into clinical trials, the last follow-up was completed in March 2017 for this analysis.

### Tumor samples

DNA was extracted from sections of the most recent FFPE tumor specimens available from biopsies or surgical resections. Optimal tumor regions were identified by clinical breast pathologists (AMM and HKB). Tumors containing a minimum acceptable tumor cellularity of 10% were processed with tumor regions isolated by 1–2 × 1 mm punch from FFPE blocks or manual macrodissection of unstained material from 15 to 20 slides. FFPE samples were deparaffinized and treated with proteinase K, followed by DNA extraction using the QIAmp DNA FFPE Tissue Kit (Qiagen, Germantown, MD) and quantification using the Qubit dsDNA Assay kit on the Qubit 2.0 Fluorometer (Thermo Fisher Scientific, Waltham, MA). DNA was extracted from peripheral blood samples for germline testing using either standard manual phenol/chloroform extraction methods or automated extraction (MagAttract DNA Mini M48 kit; Qiagen).

### Molecular profiling assays and PTEN testing

Molecular profiling was performed in a College of American Pathologists (CAP) accredited and Clinical Laboratory Improvement Amendments (CLIA) certified laboratory. Details of the molecular profiling assays have been described in detail elsewhere [8]. Briefly, three molecular profiling assays were used over the study period: the TruSeq Amplicon Cancer Panel (TSACP, Illumina) on the MiSeq sequencer (Illumina) covering hotspot regions of 48 genes; the Ion AmpliSeq Cancer Panel (ASCP, Thermo Fisher Scientific) on the Ion Proton sequencer (Thermo Fisher Scientific) covering hotspot regions of 50 genes; and a custom multiplex genotyping panel on a matrix-assisted laser desorption/ionization time-of-flight (MALDI-TOF) mass-spectrometry platform (MassARRAY, Agena Bioscience, San Diego, CA) to genotype 279 mutations within 23 genes. Specific details of the sequencing panels used are shown in Supplementary Tables 1–3. FFPE samples tested by the TSACP and ASCP panels also had testing of matched blood samples for germline mutations. Sequence alignment, base calling, and variant assessment for the TSACP and ASCP panels were as previously described [8]. The scheme of Sukhai et al. [9] was used for assessment and classification of variants.

A subset of patients enrolled had testing for phosphatase and tensin homolog (PTEN) using immunohistochemistry (IHC) with rabbit monoclonal Ab 138G6 (Cell Signaling Technology, Danvers, MA) on a Dako platform using a dilution of 1:50 and Flex + 30 protocol. Complete absence of tumor cell staining with positive staining of surrounding tumor stroma fibroblasts/endothelial cells was used to denote PTEN deficiency [10].

Molecular profiling results were included in the electronic medical record and returned to the treating oncologist. The clinical significance of profiling results was discussed with PM patients during a routine clinic visit by their treating oncologist and a PM oncologist reviewed the results with patients treated at other hospitals by telephone. Oncologists were provided with regular summary tables of testing results and mutation-specific clinical trial listings available at PM.

### Clinical data collection

For each patient, baseline patient and tumor characteristics, treatment regimen(s), time on treatment(s), and survival were retrieved from medical records and updated every 3 months. Therapeutic clinical trial enrollment was evaluated from the date of reporting molecular profiling results until April 2016. Most recent follow-up for patients enrolled in clinical trial for this analysis was March 2017. Genotype-matched clinical trials were defined as those restricting enrollment to patients with specific somatic mutations, those with a targeted drug with enriched clinical or preclinical activity in a patient’s genotype, or those involving use of a drug that inhibited a pathway directly linked to the somatic mutation. Enrollment on clinical trial was based on trial availability and patient or physician preference and did not follow a pre-specified algorithm.

### End-points

Radiological responses were defined as complete response (CR), partial response (PR), stable disease (SD), or progressive disease (PD) based on RECIST 1.1 criteria [11]. For comparison of clinical outcomes on matched versus unmatched therapies, therapeutic outcomes were evaluated according to time on treatment, defined as date of trial enrollment until date of discontinuation of investigational treatment.

### Statistical analysis

Descriptive statistics were used to summarize patient characteristics, profiling results, and therapeutic activity. A generalized estimating equation model [12] was used to compare patients with profiling results treated on genotype-matched and genotype-unmatched trials. A mixed model was used to compare time on treatment, accounting for individual patients who were included on multiple therapeutic trials [13]. Differences with *p* values of < 0.05 were considered statistically significant.

## Results

### Molecular profiling and PTEN results

**Fig. 1:**
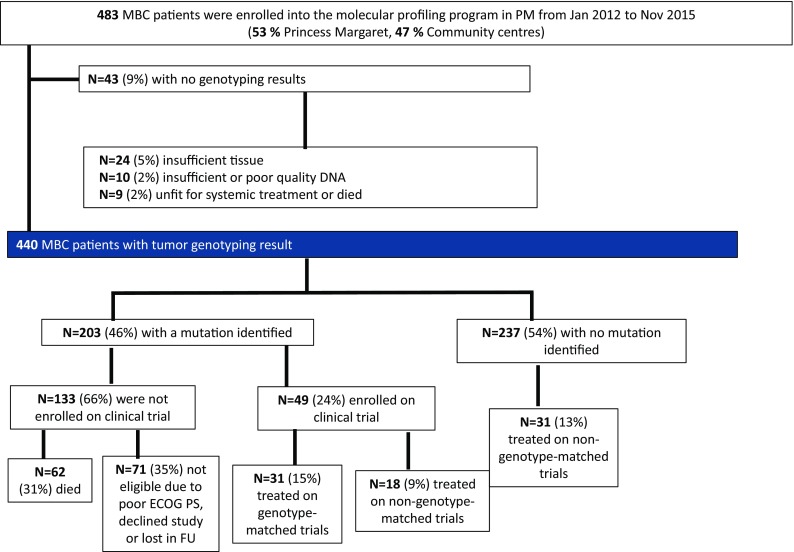
Consort diagram of study schema and genotyping results

A total of 483 patients with MBC were enrolled from January 2012 to November 2015 as outlined in Fig. 1. Fifty-three percent of patients were registered by their treating oncologist at PM and 47% were referred from local community hospitals. Forty-three patients (9%) who signed consent did not undergo profiling because of clinical deterioration before testing was performed or insufficient and/or poor quality tissue or DNA for sequencing analysis.

Of the 440 patients with genotyping results, 203 (46%) had at least one mutation identified. The median follow-up time from date of profiling was 12.5 months (range 2 days–46 months). Table 1 lists the characteristics of patients who had molecular profiling performed on their tumors. The median age of all enrolled patients was 53 years (range 20–83 years) and median lines of systemic therapy (including endocrine treatment in hormone receptor-positive patients) received before enrolling into the molecular profiling program was 2 (range 1–18). Most patients had their primary tumor profiled (*n* = 326, 74%). The most common histologies were ductal (*n* = 338, 70%) and lobular carcinomas (*n* = 24, 5%). Initial stage at diagnosis was either stage I or II in 155 patients (32%), stage III in 76 patients (16%), stage IV in 57 patients (12%). Most tumors were grades 2 or 3 (*n* = 263, 54%). Receptor status was ER positive/HER-2 negative in 265 patients (55%), HER-2 positive in 62 patients (13%), and triple negative in 91 patients (19%). Characteristics of patients with one or more mutations identified are also listed in Table 1.

**Table 1:** Baseline characteristics of all patients enrolled in the molecular profiling program

Characteristics	All patients (*n* = 483)		Patients with ≥ 1 mutation (s) (*n* = 203)		Patients with no mutations (*n* = 237)	
	Number	%	Number	%	Number	%
Median age (range)	53 (20–83)					
Histology						
Ductal carcinoma	338	70	152	75	186	78
Lobular carcinoma	24	5	12	6	14	6
Mixed ductal and lobular carcinoma	9	2	5	2	2	1
Other histology	6	1	6	3	0	0
Invasive mammary carcinoma, type not defined	64	13	28	14	35	15
Unknown	42	9	0	0	0	0
Initial stage of diagnosis						
I/II	155	32	73	36	79	33
III	76	16	34	17	42	18
IV	57	12	19	9	37	16
Unknown	195	40	76	37	79	33
Tumor grade at diagnosis						
1	14	3	7	3	7	3
2 and 3	263	54	116	57	142	60
Unknown	206	43	77	38	88	37
Median lines of systemic treatment (range)	2 (1–18)^a^					
Receptor status						
ER positive/HER2 positive	44	9	19	9	25	11
ER positive/HER2 negative	265	55	112	55	153	65
ER negative/HER2 positive	18	4	10	5	8	3
Triple negative	91	19	52	26	39	16
ER positive/HER2 unknown	10	2	5	2.5	5	2
Unknown	55	11	5	2	7	3
Site of the sample used for genotyping (*n* = 440)						
Primary	326	74	147	72	178	75
Metastasis	114	26	56	28	59	25
Platform used for genotyping (*n* = 440)						
Princess margaret sequenom solid tumor panel	317	72	59	29	155	65
Illumina MiSeq TruSeq amplicon cancer panel/proton platform (TSACP/ASCP)	123	28	139	71	83	35

^a^One patient received 18 lines of systemic therapies

**Fig. 2:**
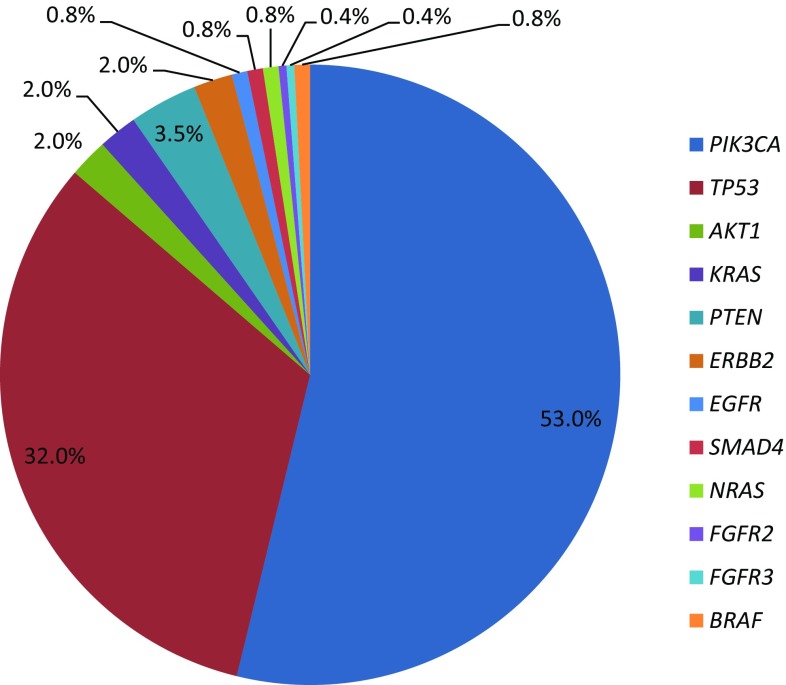
Overall frequency (percentage) of somatic mutations out of 254 total mutations identified

**Fig. 3:**
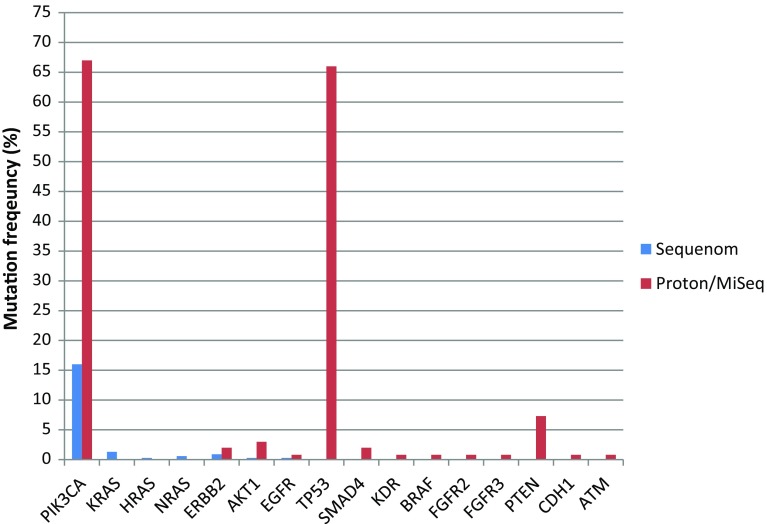
Overall frequency (percentage) of somatic mutations identified in all patients profiled (*n* = 440) on the Sequenom versus MiSeq/Proton platforms

As shown in Fig. 2, the most common somatic mutations were *PIK3CA* (*n* = 134, 53%), *TP53* (*n* = 81, 32%), *PTEN* (*n* = 9, 3.5%), and *AKT1* (*n* = 5, 2%). Other genomic alterations were less common, including mutations in *KRAS, ERBB2*, *BRAF*, *EGFR*, *SMAD4*, *FGFR1*, *FGFR2*, *FGFR3*, *HRAS*, *NRAS*, *KDR*, and *CDH1*. Details on the specific mutations identified in each patient are provided in Supplementary Table 4. Figure 3 shows mutation frequencies detected by Sequenom versus TSACP/ASCP. Table 2 lists frequencies of mutations by receptor status of the tumor tissue used for molecular profiling. *PIK3CA* was the most common mutation identified in both ER positive/HER2 negative (31%) and ER positive/HER2 positive (64%) of tumors. *TP53* mutations were the most commonly identified molecular alterations in triple-negative breast tissues at 41% and in ER negative/HER2 positive samples at 33%. PTEN expression was tested by IHC and detected in 141 out of 163 patients (87%). Twenty-two patients (14%) had loss of PTEN in their tumors and 2 of these patients with PTEN deficiency (9%) had tumors with somatic mutations in *PIK3CA*.

**Table 2:** Somatic mutation frequencies by receptor status of archival tissues used for molecular profiling

Gene	Receptor status			
	ER+/HER2− *n* = 265 (%)	ER+/HER2+ *n* = 44 (%)	ER−/HER+ *n* = 18 (%)	ER−/PR−/HER2− *n* = 91 (%)
*PIK3CA*	81 (31)	28 (64)	3 (17)	17 (19)
*TP53*	20 (8)	16 (36)	6 (33)	37 (41)
*PTEN*	3 (1)	2 (5)		2 (2)
*AKT1*	3 (1)		1 (6)	
*BRAF*	1 (0.4)			
*EGFR*	2 (0.8)			
*ERBB2*	3 (1)		1 (6)	
*FGFR2*	1 (0.4)			
*KRAS*	2 (0.8)			2 (2)
*SMAD4*	1 (0.4)			1 (1)
*KDR*				1 (1)

### Treatment for patients enrolled in clinical trials

A total of 80 patients (18%) with sequencing results were enrolled in therapeutic clinical trials at our institution, including 31 on genotype-matched trials and 49 on non-genotype-matched trials (Fig. 1). Of the 203 patients with at least one mutation, 49 (24%) were enrolled in a therapeutic clinical trial after profiling results were reported, with 31/203 (15%) enrolled in a genotype-matched trial and 18/203 (9%) treated on non-matched trials (includes patients with actionable mutations not matched to a genotype-specific trial). Of 237 patients with no mutations identified, 31 (13%) were enrolled in therapeutic trials after the date when molecular profiling results were reported.

The most common classes of drugs received on genotype-matched trials were PI3K inhibitors. Table 3 shows the somatic mutations identified and the best responses for patients treated on matched clinical trials. Radiological responses measured by RECIST 1.1 criteria are reported where available for patients treated on genotype-matched trials at our institution (including several patients enrolled in multiple matched trials). There was no significant difference for median time on treatment for patients treated on genotype-matched versus non-genotype-matched clinical trials (3.6 vs. 3.8 months; *p* = 0.89). Response assessments were compared between genotype-matched and non-genotype-matched patients enrolled in clinical trials as shown in Table 4. There were no significant differences in the best responses for patients enrolled in genotype-matched versus non-matched trials (*p* = 0.51).

**Table 3:** Genotyping results and clinical trial agents for patients on matched trials

Site of sample used for profiling	Mutations identified	Receptor status	Class of targeted drug received on clinical trials	Best response
Primary lesion	PIK3CA ≫ N345 K	ER+/HER2−	Combination with PI3Ki	N/A
Primary lesion	PIK3CA ≫ H1047R	Triple negative	Combination with PI3Ki	PR
Primary lesion	PIK3CA ≫ H1047R	ER+/HER2 unknown	Combination with PI3Ki	PD
Primary lesion	PIK3CA ≫ E545 K	ER+/HER2−	PI3Ki	SD
Primary lesion	PIK3CA ≫ E542 K	Triple negative	Combination with PI3Ki	PR
Primary lesion	PIK3CA ≫ H1047R	ER+ HER2+	Combination with PI3Ki	PD
Primary lesion	PIK3CA ≫ H1047R	ER+/HER2−	PI3Ki	PR
Primary lesion	PIK3CA ≫ H1047R	ER + HER2+	AKTi	PD
Metastatic lesion	PIK3CA ≫ Q545G PTEN ≫ L320X PIK3CA ≫ R93Q	ER+/HER2−	AKTi	PD
Metastatic lesion	PIK3CA ≫ E545 K	ER+/HER2−	PI3Ki	N/A
Primary lesion	PIK3CA ≫ H1047R TP53 ≫ P278S	ER+/HER2−	PI3Ki	SD
Primary lesion	PIK3CA ≫ H1047R	ER+/HER2−	PI3Ki	PR
Metastatic lesion	PIK3CA ≫ E542 K	ER+/HER2−	PI3Ki	N/A
Metastatic lesion	PIK3CA ≫ H1047R	ER+/HER2−	PI3Ki	N/A
Primary lesion	PIK3CA ≫ N345 K	ER+/HER2−	Combination with PI3Ki	PD
Primary lesion	PIK3CA ≫ N345 K	ER+/HER2−	Combination with PI3Ki	PD
	NRAS ≫ G12D	ER+/HER2−	Combination with PI3Ki and FGFRi	PD
			PI3Ki alone	PR
Primary lesion	PIK3CA ≫ H1047L	ER+/HER2-	Combination PI3Ki	PD
Primary lesion	PIK3CA ≫ E542 K	ER+/HER2−	PI3Ki	PD
Primary lesion	PIK3CA ≫ c. 1633G > A (p.Glu545Lys)	ER+/HER2−	AKTi	SD
Primary lesion	PIK3CA ≫ E542 K ERBB2 ≫ L755S	ER+/HER2−	HER2 TKI	PD
Primary lesion	PIK3CA ≫ E545 K TP53 ≫ L252del BRAF ≫ c.1315-4C > G	ER−/HER2+	Combination with PI3Ki	SD
Primary lesion	PIK3CA ≫ N345 K	ER+/HER2−	PI3Ki	SD
Primary lesion	PIK3CA ≫ H1047E	ER+/HER2−	PI3Ki	SD
Primary lesion	PIK3CA ≫ H1047L TP53 ≫ C238Y	ER−/HER+	Combination with PI3Ki and EGFRi	SD
Primary lesion	PIK3CA ≫ H1047R	ER+/HER−	PI3Ki	N/A
Primary lesion	PIK3CA ≫ H1047L	ER+/HER2−	PI3Ki	SD
Metastatic lesion	PIK3CA ≫ H1047R TP53 ≫ R248Q	ER-/HER-	PI3Ki	SD
Primary lesion	PIK3CA ≫ E545 K TP53 ≫ Q192X	ER+/HER2−	PI3Ki	SD
Metastatic lesion	ERBB2 ≫ D769H PIK3CA ≫ N345 K	ER+/HER2−	HER2 TKI	SD
			PI3Ki	SD
Primary lesion	FGFR2 ≫ Y376C	ER+/HER2-	FGFRi	SD

Best responses according to RECIST 1.1 criteria are listed if available. One patient was enrolled in two different clinical trials with PI3K inhibitors

*N/A* not available, *PR* partial response, *PD* progressive disease, *SD* stable disease, *PI3K* phosphatidylinositol 3-kinase, *mTOR* mammalian target of rapamycin, *AKT* protein kinase B, *EGFR* epidermal growth factor receptor, *TKI* tyrosine-kinase inhibitor, *FGFR* fibroblast growth factor receptor

**Table 4:** Comparison of best responses for patients on genotype-matched versus non-genotype-matched clinical trials

Best response	Trial enrollment		Total
	Non-genotype-matched	Genotype-matched	
No response	55	27	82
Response	6	5	11
Total	61	32	93

Response data not available for all patients. Includes two patients enrolled in multiple trials

## Discussion

In this study, we identified one or more somatic mutations in 46% of patients with metastatic breast cancer using Sequenom hotspot or small targeted NGS panels. An important feature of our study is that we included patients receiving treatment in both academic and community settings that were referred for profiling while on standard of care therapies for metastatic disease. Albeit a non-randomized comparison, we did not observe a difference in time on treatment or best responses for profiled MBC patients treated on genotype-matched versus non-matched trials.

An earlier study by Von Hoff and colleagues used IHC, FISH, and oligonucleotide microarrays for molecular profiling of tumor tissue from 66 patients (only 18 of whom had MBC) with refractory metastatic disease to match patients to specific therapies and found a longer PFS on matched therapy compared to the patient’s previous regimen [14]. However, the majority of MBC patients on this study received chemotherapy, hormonal or HER2-targeted therapies, not targeted treatments based upon identification of somatic mutations. Tsimberidou and colleagues used standard PCR-based sequencing to detect specific somatic genomic alterations in patients with refractory advanced malignancies and patients were enrolled in clinical trials according to genotype results [15]. Patients matched to targeted therapy had improved response rates, longer time to treatment failure (TTF), and longer survival than non-matched patients. However, only 16 patients in this study had MBC. In breast cancer-specific studies, SAFIR01 study used comparative genomic hybridization (CGH) array and Sanger sequencing to identify molecular alterations and match patients to targeted therapies [16]. Only 9% of patients matched to targeted therapies had objective responses to treatment. In contrast to our study, where we used archival tissues for molecular testing, SAFIR required biopsy of the metastatic site if accessible. Also SAFIR limited enrollment to patients with no more than two prior lines of chemotherapy whereas our study did not have a limit on prior number of lines of chemotherapy.

More recent studies have used new technologies, such as NGS, to identify targetable molecular alterations. In the SHIVA trial, Le Tourneau and colleagues established molecular profiles for advanced solid tumors using targeted NGS, analysis of gene copy number alterations, and analysis of hormone receptor expression by IHC to match patients to targeted therapies [17]. This study differs from ours in several important aspects. The targeted agents given to the experimental group in SHIVA were drugs approved for clinical use but outside their approved indications, while in our study we matched patients to investigational drugs available through clinical trials at our institution. SHIVA required mandatory fresh biopsies whereas we tested archival samples.

In the MOSCATO 01 Trial, patients with advanced cancers were matched to targeted therapies based on molecular alterations and 33% were shown to have improved PFS outcomes with matched therapies when compared to PFS on prior therapy. Of the entire cohort, 19% of those matched to therapies were MBC patients. Molecular alterations were identified through array comparative genomic hybridization, NGS, and RNA sequencing performed on fresh frozen tumor biopsies [18]. MOSCATO 01 differs from our study in that they required fresh biopsies, which limited the patients enrolling in study to those willing to undergo invasive testing, those with tumors accessible for biopsy, and those willing to wait for the results of their molecular profiling for a decision on subsequent treatment. In our study patients had molecular profiling done on archival tissues while they were on systemic therapy and at the time of progression could be referred back for discussion of available targeted therapies on clinical trials based on identified genomic alterations.

In our study, the most common mutation identified among patients with genotyping results (*n* = 440) was in *PIK3CA* (*n* = 123, 28%), which is consistent with other recent molecular profiling studies in MBC [16, 19–22]. Although mutations in *PIK3CA* are common in breast cancer, they have not been reliably predictive of clinical response to drugs targeting the PI3K/mTOR pathway [4–7]. *PIK3CA* mutations identified in archival tissues in BOLERO-2 [5], FERGI [23], BELLE-2 [24], and BELLE-3 [25] studies were not predictive of differential benefit with inhibitors of PI3K or mTOR. Thus in our study, where the majority of patients treated on genotype-matched trials were treated with PI3K inhibitors, the lack of significant differences in best responses and time on treatment may be reflective of the limited efficacy of PI3K inhibitors observed in *PIK3CA*-mutant metastatic breast cancers.

As an alternative to tissue biopsies, identifying key mutations in circulating tumor DNA (ctDNA) in peripheral blood samples may provide more actionable information about the molecular profile of metastatic tumors. For example, in the BELLE-2 [24] and BELLE-3 [25] studies with the pan-PI3K inhibitor buparlisib combined with endocrine therapy, there was greater magnitude of effect on progression-free survival in the *PIK3CA* mutant subgroup identified through cell-free DNA but not in the *PIK3CA* mutant subgroup identified through archival tissue samples. However, in the BOLERO-2 study which examined the benefit of everolimus, an inhibitor of the PI3K/AKT/mTOR pathway, patients benefited from the addition of everolimus to endocrine therapy regardless of the presence of a *PIK3CA* mutation in either archival tissues [5] or cell-free DNA [26]. With novel isoform-selective PI3K inhibitors currently in testing clinical trials [27, 28], it remains to be determined whether PIK3CA mutation status tested using archival tissue samples or cell-free DNA is a biomarker of treatment response.

It is important to note that FERGI, BELLE-2, and BELLE-3 all involved pan-PI3K inhibitors, while the majority of patients treated on genotype-matched clinical trials at our institution were treated with isoform-selective PI3K inhibitors. More selective, isoform-specific PI3K inhibitors may be more effective and less toxic than pan-PI3K inhibitors, potentially leading to improved therapeutic outcomes [29]. However, the number of patients treated on isoform-selective versus pan-PI3K inhibitors is too small in our study to be able to make any conclusions regarding differential therapeutic outcomes.

*TP53* was the second most common mutation identified in our breast cohort. The prevalence of *TP53* mutations is lower in our cohort compared with other reports because *TP53* mutation hotspots were not included in the Sequenom assay and the TSACP panel does not include full sequencing of the *TP53* gene. *TP53* mutations are associated with aggressive breast cancers and are identified in > 80% of basal-like breast cancer. Basho et al. analyzed archival tissues from 500 MBC patients including all subtypes using hotspot mutation testing and found that *TP53* mutations were associated with worse clinical outcomes [22]. *TP53* mutation is not currently a targetable genomic alteration, although inhibition of the protein kinase WEE1 in *TP53*-mutated cancers may be a potential future therapeutic approach [30, 31]. Additionally more rare genomic alterations such as *AKT1* and *ERBB2* mutations, each occurring in 2% of MBC patients in our cohort, are potential targets for AKT and ERBB2 inhibitors [32].

There are several important limitations to note in our study. First, we used archival tumor tissue which in some patients was many years removed from the date of study enrollment. Second, although our institution has a broad portfolio of early phase clinical trials, not all classes of targeted drugs were available during the course of the study for patients with actionable mutations. The majority of genotype-matched clinical trials available at our institution during the study period involved a PI3K inhibitor and most trials did not involve alpha isoform-selective/specific PI3K inhibitor that may have greater activity in *PIK3CA* mutant breast cancers [33, 34]. Access to genotype-matched therapies for patients with actionable mutations was limited, and the clinical outcomes reported in our study may reflect the availability of effective targeted therapies and/or the usefulness of a sequencing-based treatment strategy. Available data do not allow for the exploration of the relative contribution of these limitations on our results. Access to a greater number of drugs targeting specific molecular alterations in basket trials, which enroll patients based on the molecular alteration of the tumor not on specific tumor types, such as the NCI-MATCH program [35], would broaden the availability of targeted drugs for actionable mutations. Unlike some other types of solid tumors, MBC patients have access to many lines of systemic therapy outside of clinical trials and some may have preferred other treatment options when invited to participate in a genotype-matched clinical trial. Only a small number of patients were enrolled in clinical trials following receipt of molecular profiling results and there was significant heterogeneity in the type of matched drug treatments received. There were also limitations in our sequencing approach. We used small hotspot genotyping or targeted sequencing panels for frequent point mutations and insertions/deletions only. While these capture the majority of somatic alterations in breast cancer, we did not assess gene amplifications or fusions which are potential genomic driver or tumor suppressor alterations that are relevant for genotype-matched treatment selection, such *FGFR1* amplification or fusions, *BRCA1/2* mutations, *NTRK* fusions, *PIK3CA* fusions and *ERBB2* fusions [36].

In conclusion, our data provide “real-world” clinical outcomes for patients with MBC who have undergone molecular profiling with hotspot genotyping or small targeted NGS testing. We found that only a small percentage of patients with MBC profiled using hotspot genotyping or small targeted NGS testing subsequently enrolled in genotype-matched clinical trials. In this non-randomized comparison, we did not observe a difference in time of treatment for patients subsequently enrolled in genotype-matched versus non-genotyped-matched clinical trials. Further studies are required to establish the clinical utility of routine multi-gene mutation testing for patients with MBC.

## Electronic supplementary material

Below is the link to the electronic supplementary material.

Supplementary material 1 (DOCX 244 kb)
