# Supplementary material for: Impact of multi-gene mutational profiling on clinical trial outcomes in metastatic breast cancer
Source: Breast Cancer Res Treat. 2017 Nov 24;168(1):159–68. doi: 10.1007/s10549-017-4580-2 (PMC5847065; doi:10.1007/s10549-017-4580-2)
Supplement: Supplementary file 1 — Supplementary material 1 (DOCX 244 kb) [file 10549_2017_4580_MOESM1_ESM.docx]

**Supplementary Tables**

**Supplementary Table 1.** 279 variants in 23 genes detected by the MALDI-TOF MS assay (MassArray, Agena Biosciences)

**Supplementary Table 2.** 48 genes included in the TruSeq Amplicon cancer Panel (TSACP, Illumina), which consists of 212 amplicons covering a total genomic region of 35.84 kb.

**Supplementary Table 3.** 50 genes included in the Ion AmpliSeq Cancer Panel v2 (ASCP, ThermoFisher), which consists of 207 amplicons covering a total genomic region of 22 kb
